# Structural Stability of Titanium-Based High-Entropy Alloys Assessed Based on Changes in Grain Size and Hardness

**DOI:** 10.3390/ma16237361

**Published:** 2023-11-27

**Authors:** Dominika Górniewicz, Krzysztof Karczewski, Zbigniew Bojar, Stanisław Jóźwiak

**Affiliations:** Faculty of Advanced Technologies and Chemistry, Military University of Technology, Sylwestra Kaliskiego 2, 00-908 Warsaw, Poland; dominika.gorniewicz@wat.edu.pl (D.G.); zbigniew.bojar@wat.edu.pl (Z.B.); stanislaw.jozwiak@wat.edu.pl (S.J.)

**Keywords:** HEA, thermal stability, solid solution, laves phase, grain size, microhardness

## Abstract

The thermal stability of the grain structure and mechanical properties of the high-entropy two-phase TiCoCrFeMn alloy produced by powder metallurgy, assessed based on microhardness measurements, was analyzed in this work. For this purpose, material obtained via sintering using the U-FAST method was subjected to long-term heating at a temperature of 1000 °C for up to 1000 h in an argon atmosphere. For homogenization times of 1, 10, 20, 50, 100, and 1000 h, grain size changes in the identified phase components of the matrix were assessed, and microhardness measurements were conducted using the Vickers method. It has been shown that the changes in the analyzed parameters are closely correlated with non-monotonic modifications in the chemical composition. It was found that the tested alloy achieved structural stability after 100 h of annealing. A stable grain size was obtained in the BCC solid solution of approximately 2 µm and the two-phase BCC+C14 mixture of roughly 0.4 µm. Long-term heating for up to 1000 h caused the grain structure to grow to 2.7 µm and 0.7 µm, respectively, with a simultaneous decrease in hardness from 1065 HV to 1000 HV. The chromium and titanium diffusion coefficient values responsible for forming the BCC solid solution and the Laves C14 phase, including the material matrix, were also determined at this level to be D_Cr_ = 1.28 × 10^−19^ (m^2^·s^−1^) and D_Ti_ = 1.04 × 10^−19^ (m^2^·s^−1^), demonstrating the sluggish diffusion effect typical of high-entropy alloys.

## 1. Introduction

A critical issue in the context of materials for engineering and industrial applications is their structural stability under various operating conditions [1]. A crucial differentiation element in many HEAs (high-entropy alloys) in comparison to conventional alloys is the room temperature phase vis à vis the high-temperature phase. Conventional elements and alloys present at room temperature have a more stable, close-packed structure, whereas at high temperatures, the structure is more open. Furthermore, at lower temperatures, an ordered phase may be observed, while a disordered phase is observed at higher temperatures. This trend does not apply to all HEAs, as in some cases, the reverse trend can be noted [2].

In terms of thermal stability, an experiment of heating the FeCrNiTiAl alloy at 800–1100 °C for 144 h did not result in the formation of new phases, but only in a slight change in the Laves phase content, which suggests that high-entropy phases are relatively stable at high temperatures. At the same time, it was shown that alloys heated under these conditions had a hardness of 500 HV, which is 30 HV lower compared to the state after casting. This is because, at a temperature of 1000 °C, increased Laves precipitation appears in the alloy, which leads to a reduction in the internal elastic stresses present in the dendritic structure. In this case, the hardness of the alloy declines notably [3].

In the case of high-entropy alloys, the structural stability may be influenced by various factors, for example, the types of chemical bonds. Paper [4] examined the cast alloys Al_1_Cr_1_Fe_1_Mn_1_Ni_1_Ti_1_ and Al_1_Cr_2_Fe_4_Mn_4_Ni_1_Ti_4_, which contain bonds of various natures, i.e., metallic, covalent, and ionic. They found that different bonds are important in achieving the alloy’s high strength and chemical stability [4].

It can be assumed that the structural stability and the mechanical properties may be related to the metallic bond and covalent bond strengths in Co–Cr–Fe–Ni HEAs, which, in effect, are dependent on the element contents. The lattice distortion effect can be maximized by the chemical interactions between elements in the HEA. An increase in the lattice constant can result from the lattice distortion initiated by a growth in Cr and Ni contents or a decline in Co and Fe contents. Therefore, an increase in Co and Fe contents and a decrease in Cr and Ni contents could enhance the Co–Cr–Fe–Ni HEA’s stiffness, whereas the Ni element has a major effect on the stiffness. When combining structural stability and brittle–ductile behavior, it was found that the stiffness variation with composition is in accordance with that of structural stability but contradictory to the brittle–ductile behavior. The Co–Cr–Fe–Ni HEA examined in this research can be in a single FCC solid-solution phase, and all alloys are mechanically stable in the initial state. With an increase in the Co/Fe content and a decrease in the Cr/Ni content, the stability of the structure grows, whereas the constants of the equilibrium lattice grow with an increase in Cr/Ni content and a decrease in Co/Fe content. Moreover, the mass density grows with Co, Fe, and Ni contents and declines with the Cr content. The ductility may improve with increasing Cr/Ni contents and decreasing Co/Fe contents. The stiffness is enhanced with growing Co/Fe or declining Cr/Ni contents. Specific alloy components influence its performance in a significant way; Cr specifically impacts the structural stability, Fe affects the equilibrium lattice constant, Cr impacts the mass density, Fe influences the ductility, and Ni affects the stiffness. Research reveals that the stiffness and structural stability of Co–Cr–Fe–Ni HEAs are enhanced, while the ductility may weaken with an increase in the Co/Fe content or a decrease in the Cr/Ni content. It can be assumed that the structural stability and mechanical properties can be related to the strength of the metallic bonding as well as the covalent bonding within Co–Cr–Fe–Ni HEAs, which is a result of the change in element content [5].

The VEC (valance electron concentration) parameter is another component that affects the structural stability of HEAs. The equivalency of alloying elements in stabilizing FCC- or BCC-type solid solutions in HEAs may indicate a correlation between this equivalency and the electron concentration effect in solid-solution-forming HEAs. The stabilization of the BCC phase occurs at a lower VEC, while the stabilization of the FCC phase occurs at a higher VEC. FCC, as well as BCC, phases occur at intermediate VECs. Almost quantitatively, FCC phases exist at VECs ≥ 8.0, BCC phases exist at VECs < 6.87, and a mixture of FCC and BCC phases exists at 6.87 ≤ VECs < 8. There are exceptions to note, which particularly refer to Mn-containing HEAs. The VEC rule offers a suitable approach to developing FCC- or BCC-structured HEAs, which primarily contain transition metal elements from the electron concentration perspective. Its validity has been proven extensively by further experiments conducted after its initial publication [6].

Well-known additional effects of HEAs also contribute to their stability—a high mixing entropy and the effect of crystal lattice distortion. In particular, they are only present in four- or five-component BCC alloys and all FCC alloys [7]. Although the high-entropy effect, which is the basis for the definition of a new group of materials, is often discussed, it has a positive effect on the tendency to stabilize solid solution phases. The idea of alloy additions is associated with additional, synergistic effects, which are visible in the phenomena observed in high-entropy alloys [8].

The occurrence of four characteristic effects also affects their properties, and, for example, a locally occurring distortion of the crystal lattice does not significantly affect the lattice parameters and bulk modulus [7]. For example, adding Mn as a fifth element to a mixture composed of Co, Cr, Fe, and Ni will lead to increased lattice distortion because the atomic diameter of this element is larger than that of the other four elements. Nevertheless, this structural effect should result in a decrease in the stability of HEA when changing from a four- to five-component system, while the entropy, on the contrary, increases, stabilizing the phase structure [9].

Due to the effect of crystal lattice distortion, the phase structure of high-entropy alloys can be considered intermediate, between stable crystalline phases with a small proportion of defects and metastable metallic glasses in which there is no long-range order. The prospect of the specific structural features in HEA—low values of diffusion coefficients, corrosion resistance, and increased plasticity at low temperatures—makes the phase stability of high-entropy alloys a priority regarding the suitability of materials in many technologies. Nevertheless, in practical applications, the metastability of the material does not constitute an obstacle when the conditions for a metastable state are specified [9].

The stability of the alloy also depends on the value of the Gibbs free energy (isobaric-isothermal potential):

G = H − S

H is enthalpy, T is the absolute temperature, and S is entropy. According to the Gibbs free energy theory, to maintain the system’s stability, the enthalpy and entropy of the mixture need to be correlated. The entropy of the mixture plays a leading role in minimizing the Gibbs free energy of the system at high temperatures [9]. There is evidence in thermodynamics that the system is in stable thermodynamic equilibrium when the pressure and temperature are constant and when G is at a minimum value. At temperature T, an entirely disordered solid solution is stable when the condition of any hypothetical change in its structure and phase is present [10]. For a disordered solution, the entropy of mixing significantly expands the dissolution range of a complex solid solution or intermetallic compound, thereby creating a simple solid solution [9]. Due to the compositional complexity of HEA alloys, one of the most important factors influencing the structural stability at high temperatures is the so-called slow diffusion effect. It is believed that slow diffusion is responsible for the thermal stability, the slowed grain growth, and the formation of nanoprecipitates [9,11,12]. It is known that the high thermal stability combined with the ability to create an amorphous form and limited diffusion kinetics predispose HEA to diffusion barriers applications. For example, Tsai et al. [13] proposed the HEA AlMoNbSiTaTiVZr alloy for use as a thin foil diffusion barrier in copper metallization. Thanks to this treatment, the high-entropy foil prevented the formation of copper silicide at temperatures up to 700 °C. The diffusion barrier layers are required to have an excellent chemical and structural stability at high temperatures due to the high diffusivity of these elements at such temperatures. Moreover, they should be as thin as possible. Since the grain boundaries of crystalline materials provide fast diffusion paths for copper, it is required that the barrier layers have an amorphous structure. The use of metallic diffusion barriers for Cu metallization in microelectronics is difficult compared to ceramic barriers due to their poorer thermal stability. However, high-entropy materials, such as the NbSiTaTiZr layer between Cu and Si [14], are good candidates for these applications due to their structural and chemical stability at high temperatures.

For example, stability within a wide temperature range is important when using piezoelectric materials as actuators. Depending on the temperature (low/high) and the environment, stability may be lost, which, as a consequence, could lead to the deterioration of the functional properties over time [15].

This article will investigate the impact of the structural stability of titanium-based high-entropy alloys by carrying out a long-term homogenization process lasting up to 1000 h of heating. This will allow for the assessment of the heat treatment that affects the growth of the granular structure.

## 2. Materials and Methods

The powder metallurgy method was used to produce the two-phase HEA alloy. This method ensures precise control over the chemical composition of the produced material. A mixture of starting powders of Ti, Co, Cr, Fe, and Mn (Table 1), with an average particle size below 45 µm, was mechanically synthesized by the dry method for 5 h in an argon atmosphere using a Fritsch Pulverisette 7 Premium planetary ball mill (Fritsch International, Idar-Oberstein, Germany) with 10 mm 100Cr6 bearing steel balls in a ratio of 8:1.

The synthesized powder mixture was sintered using the modified Spark Plasma Sintering (SPS) method, i.e., U-FAST. U-FAST and SPS apparatuses are based on the same technical principles but differ in design. This approach allows ultra-fast heating and subsequent sintering of a fine-grained structure. This kind of structure is desired concerning the Hall–Petch relationship, so the strength properties of the formed material are enhanced [16]. The densified material was subjected to microsecond pulses of ~2000 A, a pressure force of 13 kN, and a heating rate of 50 °C·min^−1^ to consolidate the powder mixture. The process of sintering was conducted at 1050 °C for one minute. Following that, sinters with a diameter of 20 mm and a height of 10 mm were strengthened in an argon protective atmosphere at 1000 °C for 1000 h to review the stability of the phase and grain structure. Then, metallographic samples for establishing the structure size were ground on SiC sandpaper (grit of 600 ÷ 4000) and polished with the use of cloths, a diamond suspension (3 µm and 0.25 µm) and a silica suspension (0.1 µm). The observation of the alloy structure was conducted on a Quanta 3D FEG Scanning Electron Microscope (SEM) (Field Electron and Ion Company, FEI, Hillsboro, OR, USA), which enabled chemical composition analysis with an energy-dispersive X-ray spectroscopy (EDS) detector and additional microdiffraction with an electron diffraction detector (EBSD). By means of X-ray diffraction (XRD) on a Rigaku Ultima IV X-ray diffractometer (Rigaku, Tokyo, Japan) with a Co lamp (λ = 1.79 Å) and on a Seifert XRD 3003 TT diffractometer (Seifert, Mannheim, Germany) with a Cu lamp (λ = 1.54 Å), a thorough phase analysis was conducted to detect reflections from different radiation absorptions. Based on the obtained XRD patterns, the share of individual phase components was analyzed using the external standard (RIR) method. Microhardness measurements were also carried out using the Vickers method with a load of 0.025 kgf to 1.0 kgf. The obtained results allowed us, through empirical models H-K [17,18,19,20], PSR [20,21,22], MPSR [20,23], and N-G [24,25,26], to determine the actual value of the tested alloy as regards microhardness measurements. At the same time, a detailed analysis of the model’s accuracy was carried out using measures based on certified hardness standards, and it was found that the PSR model had the slightest error. Due to the complexity of the above methodology, it will be discussed in a separate work devoted only to this issue.

## 3. Results

**Figure 1:**
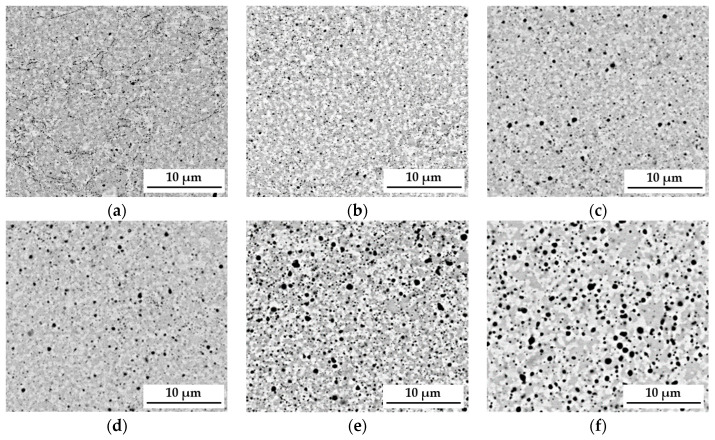
View of the two-phase grain structure of the CoCrFeMnTi alloy (SEM-BSE) for the analyzed heat treatment states: after the sintering process (**a**) and after homogenization at 1000 °C for 1 h (**b**), 20 h (**c**), 50 h (**d**), 100 h (**e**) and 1000 h (**f**).

The sintering process and long-term homogenization at a temperature of 1000 °C were applied for up to 1000 h, followed by the observation of the material with a scanning electron microscope SEM-BSE, which enabled us to derive the conclusion that the matrix structure of the analyzed TiCoCrFeMn sinter consisted of two phases, differing in grayscale (Figure 1). An analysis of changes in the chemical composition after different annealing times supported by a detailed XRD and EBSD phase investigation presented in [27] allowed us to state that the sinter matrix was composed of a BCC solid solution with a lattice parameter a ≈ 8.9 Å and a hexagonal lattice Laves C14 phase characterized by a c/a ratio of approximately 1.63. It should also be emphasized that between 10 and 100 h of annealing, intensive changes in the chemical composition were observed in the cited work, leading to the redistribution of alloy components and consequently to the formation of a phase-stable matrix. This caused significant changes in the morphology of the granular structure. After just 1 h of heating, the evident formation of the bright areas of the BCC solid solution in the two-phase BCC+C14 matrix could be observed (Figure 1b).

The further extension of the heating time to 100 h (Figure 1c–e) resulted in a noticeable expansion of both analyzed areas and allowed for achieving their complete stabilization. However, the 1000 h heating treatment (Figure 1f) did not cause further changes in the morphology and size of the grain structure of the tested high-entropic TiCoCrFeMn alloy that would be noticeable within the used scale. In addition to changes in grain size, a significant increase in the share of oxides was observed. They most likely originated from the surfaces of the starting powder particles, which were not reduced before the sintering process. Moreover, the compaction process was carried out in the air, which most likely led to the encapsulation of a certain amount of oxygen in the spaces between the powder particles. The process of homogenization under a protective argon atmosphere caused the coagulation and growth of oxide phases in the sinter structure, which can be observed in the micrographs shown in Figure 1.

**Figure 2:**
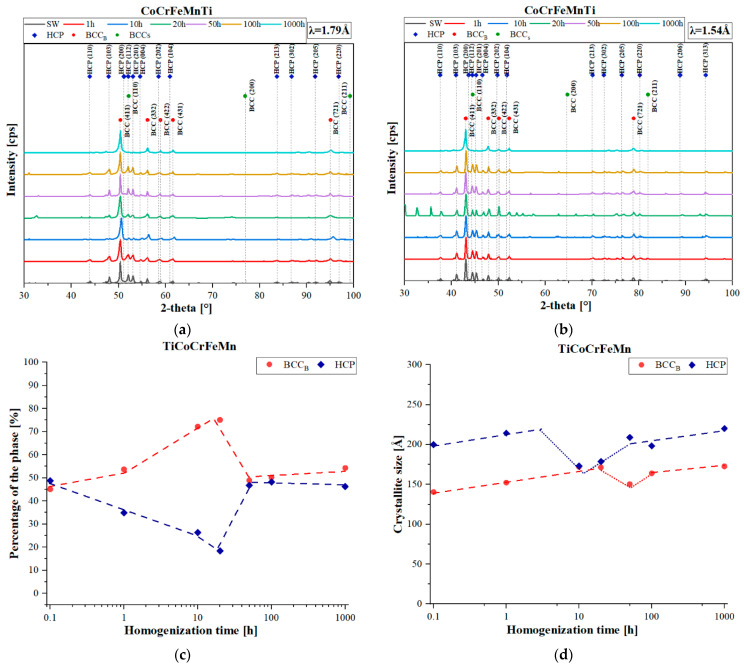
XRD patterns of the TiCoCrFeMn alloy produced by heating for various times at 1000 °C obtained with the radiation XCo—λ = 1.79 Å (**a**) and XCu—λ = 1.54 Å (**b**); the share of matrix phases determined using the RIR method (**c**); crystallite sizes of identified phases (**d**).

The quantitative analysis of changes in the primary phases, BCC or HCP (C14 Laves phase), carried out by comparing the obtained XRD reflections (Figure 2a,b) to the external RIR (Reference Intensity Ratio) standard, showed that in the initial annealing period, 1–50 h, an increase in the share of the solid solution occurred (Figure 2c). Then, as a result of the rapid fluctuations in the chemical composition, as well as the non-monotonic changes in the chromium diffusion coefficient in the solution [27], an increase in the share of the hexagonal structure of the Laves accompanied by a decrease in the content of the solution in the time interval of 50–100 h was observed. Further homogenization caused a very slow increase in the solid solution content. Also, the changes in the sizes of crystallites as a function of the homogenizing annealing time were characterized by logarithmic growth (logarithmic scale on the time axis), characterized by the rapid growth of crystallites in the initial period of the thermal treatment, and decreasing with the extension of the process time (Figure 2d). Nevertheless, as in the case of the participation of phases in the chemical composition fluctuations, the diffusive transport of atoms introduced structural defects, causing a noticeable reduction in coherent scattering within 10–100 h of annealing. After this stage, the crystallite growth returned to the initial state of logarithmic change, resulting in the Laves phase crystallites being approximately 50 µm larger.

**Figure 3:**
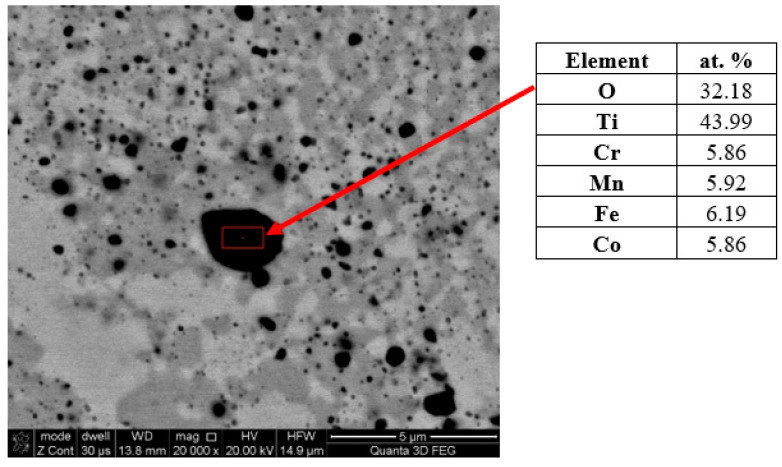
EDS surface analysis in the micro-area of oxide occurrence in the TiCoCrFeMn alloy heated for 50 h.

However, observations carried out using scanning microscopy (Figure 1) only allow for determining the change in morphology and growth, especially for the longest annealing time of 1000 h (Figure 1f). However, it should be noted that with the extension of the homogenization time, despite the process being carried out in a protective argon atmosphere, numerous coagulating and growth precipitates appeared in the matrix structure of the tested TiCoCrFeMn alloy. The analysis of the chemical composition of EDS in the selected area of occurrence of a large, relatively easy-to-interpret precipitate, presented in Figure 3, showed a significantly increased share of titanium and oxygen compared to other elements, suggesting that it was titanium oxide. Unfortunately, the size of the analyzed object means that the obtained analysis result was distorted by the interaction of the elements from neighboring areas, preventing a precise determination of the stoichiometry of the analyzed phase based on the obtained atomic share of the analyzed elements.

**Figure 4:**
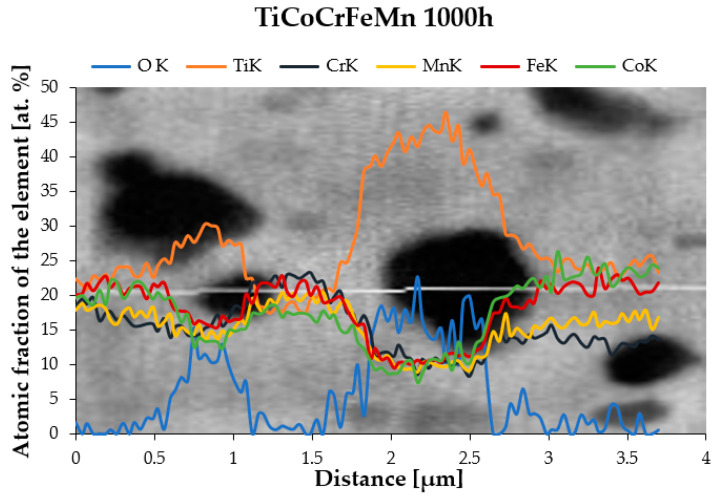
Linear EDS analysis was carried out through the areas of titanium oxide occurrence in the TiCoCrFeMn alloy heated for 1000 h.

Attempts were made to indirectly determine the chemical composition of much finer precipitates using linear analyses, indicating an increased share of titanium and oxygen in the electron beam transition zone. Such an exemplary analysis, presented in Figure 4, was carried out on the TiCoCrFeMn base alloy after annealing for 1000 h due to the stabilization of the chemical composition. Also, in this case, despite analyzing the precipitate with a diameter of approximately 0.5 µm, a significant increase in the share of titanium and oxygen was found with a simultaneous decrease in the shares of other elements, which suggests that the observed precipitates that grew with homogenization time were titanium oxides.

**Figure 5:**
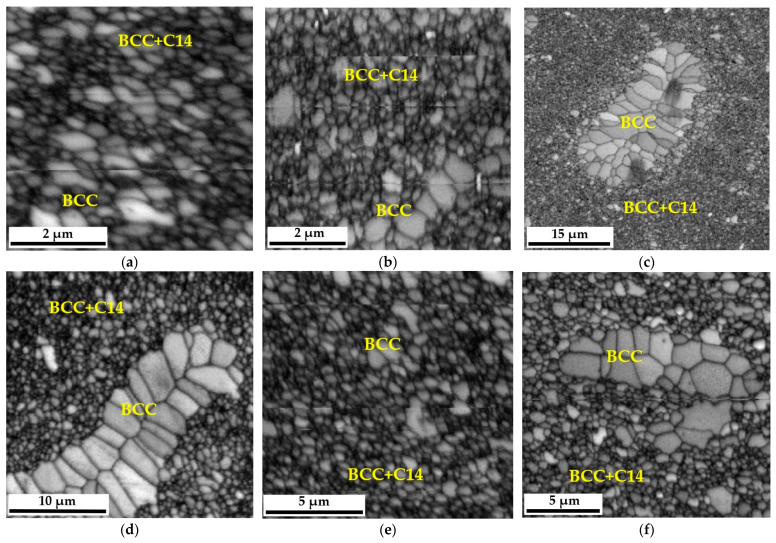
Image of the granular structure of the TiCoCrFeMn alloy made using EBSD diffraction for the analyzed heat treatment states: after the sintering process (**a**) and homogenization at 1000 °C for 1 h (**b**), 20 h (**c**), 50 h (**d**), 100 h (**e**) and 1000 h (**f**).

The changes in the morphology of the grain structure are presented in Figure 1. However, due to the use of a specific SPS sintering method, which most often leads to an ultra-fine-grained structure of sinters [28,29], the produced high-entropy material was characterized by the size of the grain structure of individual phase components, which was impossible to determine using classic microscopic methodologies such as optical microscopy or even scanning electron microscopy in the SE or BSE mode. Therefore, EBSD microdiffraction was used to reveal changes in the size of this morphological parameter, and images of the polycrystalline structure, both in the single-phase areas of the BCC solid solution and in the two-phase zones of coexistence of the BCC solid solution and the hexagonal Laves C14 phase, are presented in the form of “Image Quality—IQ” maps in Figure 5.

Grain distribution maps obtained using EBSD diffraction allowed for a quantitative assessment of the size of individual polycrystals. Nevertheless, the program used to analyze the results obtained from microdiffraction EBSD—TSL OIM Data Collection, despite being equipped with a module allowing automatic determination of grain size, is due to the specificity of the analyzed materials, consisting in the coexistence of single-phase areas of the BCC solid solution, two-phase zones BCC+C14 and precipitates. Oxides did not allow for a proper analysis of the grain size changes of individual phases forming the matrix of the produced alloy. Therefore, this analysis was performed in a non-automated mode. Based on previous chemical composition studies and the knowledge of the shades of grayscale obtained in the BSE image, specific, identifiable grains were assigned to individual phases. Then, they were outlined in the NIS Elements program, in which the microstructure image was previously scaled using the scale from SEM photos—EBSD.

**Figure 6:**
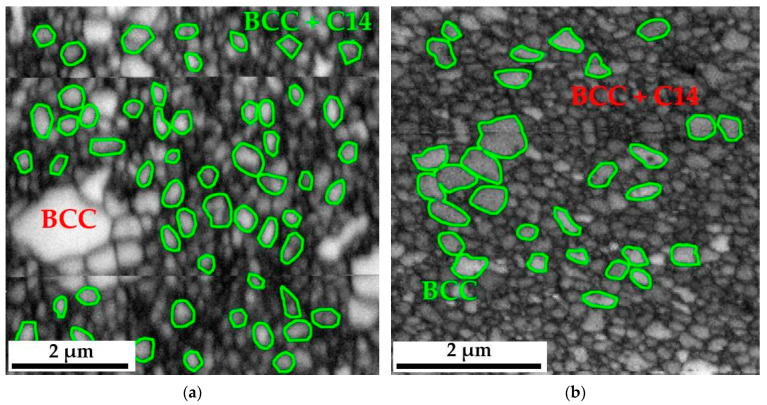
Example of grain contouring in the NIS—Elements program in the two-phase BCC+C14 area for the TiCoCrFeMn alloy after preliminary sintering (**a**) and in the single-phase BCC area for the TiCoCrFeMn alloy after 20 h of heating (**b**).

In most cases, these grains, characterized by a much larger size concerning the surrounding matrix, had a polycrystalline structure of the BCC solid solution. In the case of the two-phase region, due to the ultra-fine-grained structure, it was unfortunately impossible to distinguish which grains came from the solid solution and which from the intermetallic phase. Therefore, they were analyzed as a common two-phase region. The measurements were made in several random areas of the tested samples for at least a thousand measured objects, which provided the appropriate statistics. Example images showing how grains are outlined in the single-phase and two-phase regions are shown in Figure 6. According to Figure 5, it should be noted that in the structure of the obtained sinter, there are local areas of the BCC solid solution with a noticeably larger grain size. However, these zones become defragmented and disappear with the extension of the annealing time and the redistribution of the alloying elements [27]. Therefore, only statistically significant areas of the fine-grained BCC solution and the ultra-fine-grained mixture of the BCC solution and the Laves C14 phase were analyzed for grain size.

**Figure 7:**
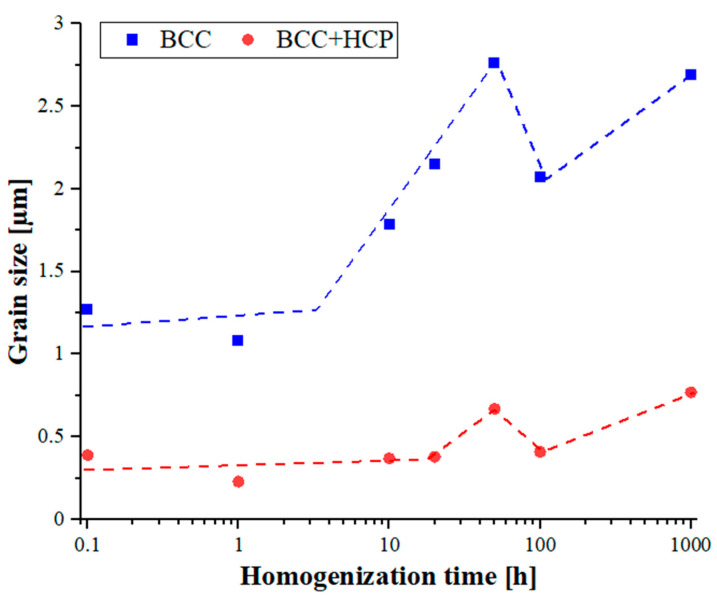
Changes in grain size in the area of the BCC solid solution and the BCC+C14 mixture of the high-entropy TiCoCrFeMn alloy matrix as a function of the homogenizing annealing time carried out at a temperature of 1000 °C.

The grain size values estimated according to the above-described methodology are summarized in Table 2 and Figure 7. In the analysis of the obtained results, it can be noticed that as in the case of previous studies on other parameters, such as changes in chemical composition [27], the grain size was also a derivative of the structural rebuilding of the produced alloys that took place during annealing. During the initial annealing period, comprising up to approximately 2 h of the process, in the structure inherited from the mechanical alloying and sintering process, a local redistribution of alloy components took place based on the original grains formed after sintering, leading to the formation of high-entropic crystal lattices of individual phase components of the alloy. Then, in the solid solution, which was rebuilt in terms of its chemical composition and lattice parameters, rapid grain growth occurred, observed until approximately 50 h of homogenization. In a further stage, a local redistribution of alloying elements took place in the solid solution grains, creating thermodynamically stable areas of the Laves phase, which resulted in a noticeable reduction in the solution grains. In the following long-term homogenization stage, the resulting stable solution structure only underwent slow grain growth, mainly due to the decline in the grain boundary’s energy. In the two-phase area, much slower grain growth was observed, caused by the coexistence of the more thermodynamically stable Laves phase in the structure of the areas, inhibiting the growth of the solid solution. As a result of the thermal treatments, the structures of the tested materials, after 1000 h of homogenization, mainly had a two-phase matrix structure with a grain size of approximately 0.77 µm, with local solution areas with a grain size of roughly 2.69 µm (Table 2).

**Figure 8:**
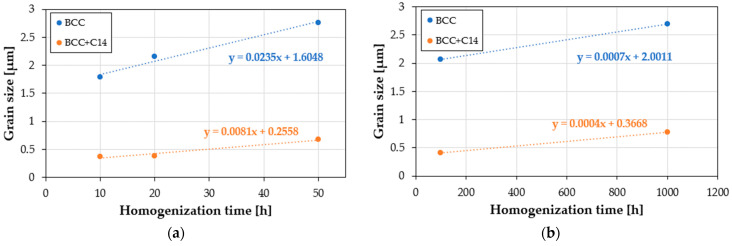
Changes in grain size in the area of the BCC solid solution and the BCC+C14 mixture of the high-entropy TiCoCrFeMn alloy matrix as a function of the homogenizing annealing time carried out at a temperature of 1000 °C after 50 (**a**) and after 100–1000 h of annealing (**b**).

The grain growth rate changed as a function of the homogenization time, presented as slope coefficients of the lines in Figure 8 and Table 3 with the most intense grain growth, characterized by a speed of 0.0235 µm·h^−1^. The upward trend was also maintained in the two-phase BCC+C14 structure initially formed during the mechanical alloying and sintering processes; however, the growth in this area was almost an order of magnitude slower, and was caused by the presence in the two-phase area of a much more thermodynamically stable hexagonal intermetallic phase with a mixed, metallic-covalent nature of bonds. After non-monotonic changes in the chemical composition, the thermodynamically stable phase structure obtained in the homogenization time interval of 100–1000 h was also characterized by a very high stability of the granular structure. In both observed phase areas of the sinter matrix, when heated at a temperature of 1000 °C for times ranging from 100 to 1000 h, the average sizes of the matrix grains increased by several hundred picometrics per hour. Such a high stability of the grain structure of the produced materials is indirect evidence confirming the slow diffusion effect or the cocktail effect in high-entropy alloys, including the tested TiCoCrFeMn alloy.

**Figure 9:**
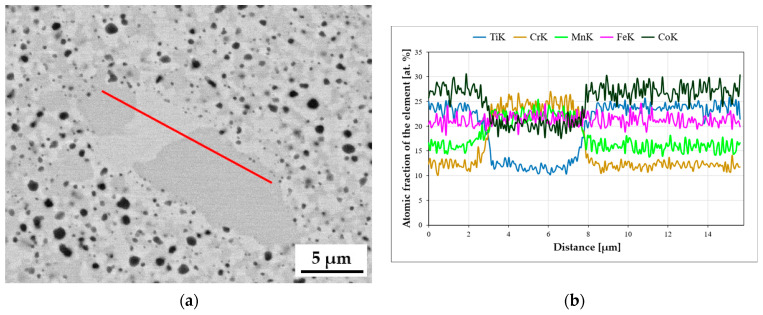

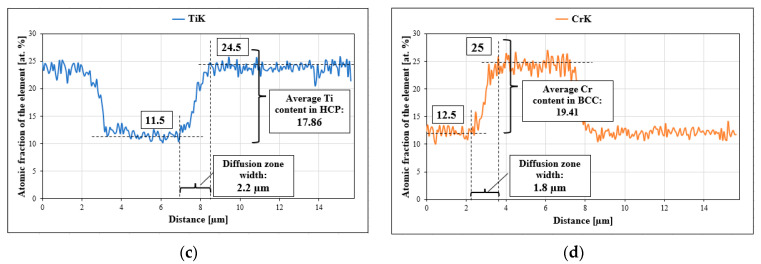
An example of a two-phase area (**a**) subjected to a chemical composition analysis along the red line marked in (**a**), (**b**) to determine the characteristics enabling the determination of the diffusion coefficient of Ti in the Laves phase (**c**) and Cr in solid solution (**d**) in a TiCoCrFeMn alloy homogenized for 1000 h.

To confirm the suggestions regarding the influence of the slowed diffusion effect in the tested high-entropy TiCoCrFeMn alloy on a noticeable reduction in the grain growth rate stabilized in terms of chemical composition and phase structure of the matrix, an analysis of changes in the diffusion coefficient of two basic elements, chromium and titanium, responsible for the formation of the BCC solid solution and the Laves phase, was carried out [27]. For this purpose, the areas of occurrence of both phases with a visible interphase boundary were isolated, on which linear analyses of the chemical composition were performed using the EDS method, focusing on the changes in the shares of chromium and titanium, taking into account the following: for titanium, the stage of transition from a solid solution to the intermetallic phase, and for chromium, diffusion from the Laves phase into the solid solution, which allowed for the analysis of the process of diffusion of the two elements into the phases stabilized by them. An example of a linear analysis characterizing the described mass transport phenomena is shown in Figure 9.

For all homogenization times, based on the analyses of the chemical composition changes, the diffusion path and the average concentration of the element in the diffusion zone were determined (Figure 9c,d). Using Equation (1) [30], the values of the diffusion coefficient D of chromium in the BCC solid solution and titanium in the Laves C14 phase were determined.

(1) $$D_{B}=\frac{x^{2}}{4*{\ln C}_{B}*t}$$

where the following holds:

*t*—diffusion duration (s);

*x*—width of the diffusion zone (m);

*C_B_* (*x*)—concentration of component *B* at distance *x*;

*D_B_*—diffusion coefficient of component *B* (m^2^·s^−1^).

**Figure 10:**
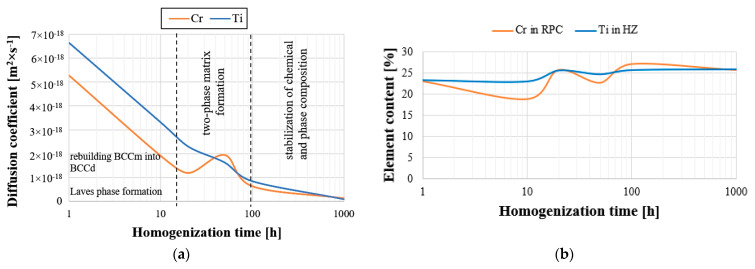
Summary of diffusion coefficients of chromium and titanium (**a**) and the share of Cr in the BCC solid solution and Ti in the Laves C14 phase, based on the analysis of the chemical composition EDS (**b**) as a function of heating time.

The summary of the determined values of the diffusion coefficients presented in Figure 10, and Table 4 shows that the values of this parameter decreased with the increase in the alloy annealing time, reaching the level of approximately 1 × 10^−19^ (m^2^·s^−1^) with the maximum annealing time. Moreover, the value of this parameter for both analyzed elements reflects the tendency of chromium to change the chemical composition in the BCC solid solution and titanium in the hexagonal Laves C14 phase (Figure 10b). Both the share of Ti in the HCP lattice and the diffusion coefficient of this element are higher and more stable than those of chromium in a solid solution. At the same time, the largest fluctuations of the coefficient were observed in the range of 10 to 100 h of heating, which were directly related to the observed fluctuations in the chemical composition. In the homogenization time interval, the greatest non-monotonic changes in the chemical composition occurred. The rapid “mixing” of ingredients was simultaneously associated with an increase in the diffusion coefficient, mainly of chromium, which accelerated the mass transport phenomena, thus stabilizing the chemical composition at this stage and phase of the tested high-entropy alloys. This allowed us to suggest that, in the initial homogenization period lasting up to 10 h of annealing, the process of forming the BCC solid solution and the densely packed, hexagonal Laves phase took place, which then progressed within 10–100 h of annealing into turbulent mass transport, resulting in the formation of a two-phase material matrix, evidenced by significant fluctuations in the chemical composition leading to chemical and phase stabilization of the material matrix during the subsequent long homogenization times.

Another purpose of determining the values of the chromium and titanium diffusion coefficients in the tested alloy was to compare them with the diffusion coefficients of these elements determined for conventional materials, and to confirm the possible effects of slow diffusion occurring in high-entropy alloys, according to most scientists. For this purpose, a literature review was performed; Table 5 lists the diffusion coefficients of chromium, and Table 6 lists those of titanium in classic materials, determined for a temperature of 1273 K, comparable to the homogenization temperature of the tested material. Among the presented data, we see a comparison of the determined values of the diffusion coefficient obtained for Cr in a solid solution and Ti in the Laves phase, which are phase components of the produced high-entropy alloy, with the diffusion coefficients of these elements in the Ni_3_Al superstructure deserving special attention. It turns out that the values obtained for the high-entropy material designed in this work in the most stable structural state (100–1000 h of heating) are characterized by a chromium diffusion coefficient value that is three orders of magnitude lower compared to the extremely thermally stable material, which is the heat-resistant Ni_3_Al phase used as a material for constructing, among others, turbine blades of jet engines. In the case of titanium, the diffusion coefficient in the high-entropic Laves phase was smaller than that in Ni_3_Al. This suggests that the slowed diffusion in the designed material, by ensuring high-temperature phase stability, allows for the potential applications of these materials as heat-resistant alloys.

**Figure 11:**
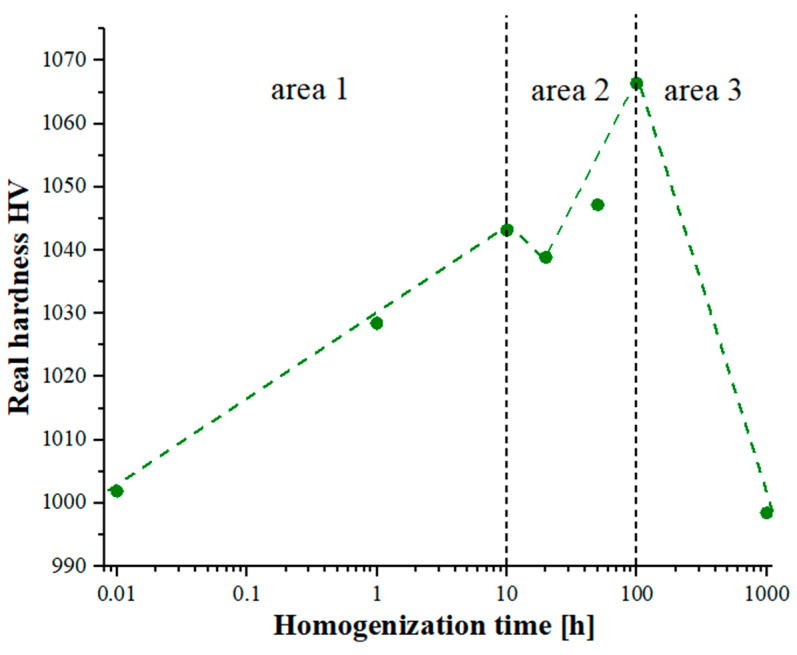
Changes in the actual hardness value as a function of the homogenization time of the tested high-entropy alloy determined from microhardness measurements based on the PSR model.

The non-monotonic changes in the chemical composition affecting the phase transformation of the produced high-entropy alloy, combined with the observed effects of slowed diffusion, in addition to affecting the growth rate of the granular structure of the matrix, also affected changes in the mechanical properties determined by hardness measurements using the Vickers method. When analyzing the changes in hardness presented in Figure 11, it is also worth noting that the changes in hardness correlated closely with the changes in the chemical composition, affecting the phase reconstruction and the changes in the Cr diffusion coefficients of the Ti solid solution in the Laves phase. For a homogenization time of up to 10 h, an increase in the hardness value was observed due to the formation of matrix phases. In the next time interval, 10–100 h, intense fluctuations in the chemical composition related to the formation of the final two-phase matrix also influenced non-monotonic changes in hardness. Continuing the heat treatment process affected the growth of the granular structure of the formed phases, resulting in a slight decrease in the hardness value.

The measurements of hardness and grain size in both areas are related to the commonly used Hall–Petch relationship, which, by definition, is the relationship between the yield strength ($\sigma_{f})$ and the grain size (*d*) (2) [38,39,40],

(2) $$\sigma_{f}=\sigma_{y}+k_{y}d^{-1/2}$$

where the following pertains:

$\sigma_{y}$—yield point of the easiest slip system in a single crystal;

$\sigma_{f}—$yield strength of the polycrystalline structure;

$k_{y}—$constant related to the strengthening of the material with grain boundaries.

**Figure 12:**
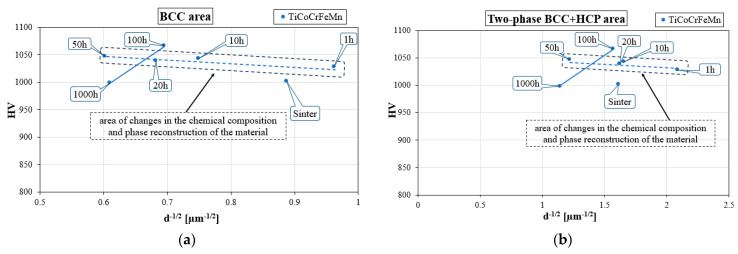
Hall–Petch relationship HV = f(d^−1/2^) determined for the TiCoCrFeMn alloy in the single-phase BCC (**a**) and two-phase BCC+HCP (**b**) regions.

This is also valid in the case of changes in the material hardness caused by changes in the grain size. Linking hardness measurements with changes in the grain size that occurred during the homogenization process allowed for the analysis of the impact of the measured morphological parameter on changes in the mechanical properties determined using hardness measurements, which is here presented via a graphical representation of the Hall–Petch relationship in Figure 12. For homogenization times of up to 50 h in both analyzed areas of the structure, as well as the solution and the two phases, the Hall–Petch relationship showed a tendency opposite to the classical one, typical for nanostructures, reducing the hardness with decreasing grain size. However, despite the obtaining of an ultra-fine-grained structure, especially in two-phase areas, this did not occur in the analyzed case. It should be remembered that the Hall–Petch relationship applies to a phase and chemically stable granular structure, while for annealing time up to 50 h, intensive phase reconstruction processes occurred, associated with non-monotonic changes in the chemical composition. Therefore, the observed “seemingly inverse” Hall–Petch effect was not dependent on the grain size. In fact, it was primarily the result of momentary changes in the mechanical properties of the polycrystalline structure caused by changes in the chemical composition and phase structure.

Only the stabilization of chemical and phase composition, which took place after 100 h of homogenizing annealing, allowed for the determination of the relationship between changes in the grain size and changes in the hardness, and the conclusion that the produced material, despite the ultra-fine-grained granular structure, had a classic Hall–Petch relationship. Taking into account the time during which grain growth occurred and the associated slight decrease in hardness, it can be concluded that the alloys produced were characterized by high structural stability combined with the stability of the mechanical properties determined via hardness tests.

## 4. Conclusions

The analysis of changes in the morphology of the grain structure of the high-entropy TiCoCrFeMn alloy carried out at a temperature of 1000 °C for up to 1000 h allowed for the following conclusions:

1. Non-monotonic changes in the chemical composition, occurring most intensively within 10–50 h of homogenization, determined changes in the tested sinter’s phase structure, influencing changes in the grain size and changes in the mechanical properties, which stabilized only after 100 h of annealing;
2. During the initial annealing period, the diffusion processes caused the formation of a BCC solid solution in the alloy matrix, based on which, after approximately 20 h of homogenization, titanium-rich areas of the Laves C14 intermetallic phase were formed. A thermodynamically stable two-phase structure was obtained after approximately 50–100 h of homogenization;
3. The observed mass transport phenomena also determined the variability in the growth of the granular structure of the matrix. The small observed areas of single-phase solid solution, inherited from the sintering process during intense, non-monotonic changes in the chemical composition occurring in the 10–50 h homogenization period, resulted in the highest growth rate of the granular structure, amounting on average to approximately 0.0235 µm·h^−1^. In the two-phase area, however, due to the formation of much more stable intermetallic Laves phase, the growth of the two-phase matrix being formed was much slower, at a rate of 0.0081 µm·h^−1^. This effect also resulted in a grain size in the two-phase region that was smaller by an order of magnitude;
4. The stabilization of the alloy, manifested by the formation of a thermodynamically stable phase structure in the matrix, also caused the stabilization of the grain structure, characterized by a slight grain growth of 0.0004–0.0007 µm·h^−1^;
5. The performed hardness measurements showed that only the stable structure of the tested high-entropy TiCoCrFeMn alloy, correlated with the size of the granular structure of the matrix, was subject, despite the ultra-fine-grained structure, to the classic Hall–Petch relationship, indirectly proving the need for long-term (100 h) heating at a temperature of 1000 °C to obtain a stable material matrix. The above statement is also confirmed by the values of the diffusion coefficients of chromium and titanium, which stabilized at these homogenization times, reaching a value confirming the occurrence of the slowed diffusion effect hat is typical for high-entropy alloys.

## Figures and Tables

**Table 1:** The chemical composition of the high-entropy alloy in question is given in atomic percent.

Element	Ti	Co	Cr	Fe	Mn
At. %	23.6	19.1	19.1	19.1	19.1

**Table 2:** Changes in grain size occurring during the homogenization process of the TiCoCrFeMn alloy carried out at a temperature of 1000 °C in the areas of the BCC solid solution and the ultra-fine-grained area of the two-phase mixture of the solid solution and the Laves phase.

		Grain Size (µm)						
Alloy	Area	Sinter	1 h	10 h	20 h	50 h	100 h	1000 h
TiCoCrFeMn	BCC	1.27	1.08	1.783	2.15	2.76	2.07	2.69
	BCC+HCP	0.39	0.23	0.37	0.38	0.67	0.41	0.77

**Table 3:** The grain growth rate of the TiCoCrFeMn alloy in the single-phase and two-phase areas.

		Grain Size Growth Rate (µm·h^−1^)	
Alloy	Homogenization Rate	BCC Area	Two-Phase Area BCC+HCP
TiCoCrFeMn	10–50 h	0.0235	0.0081
	100–1000 h	0.0007	0.0004

**Table 4:** Summary of diffusion coefficients of Cr in solid solution and Ti in the intermetallic phase.

Homogenization Time [h]	D_Cr_ (m^2^·s^−1^)	D_Ti_ (m^2^·s^−1^)
1	5.26 × 10^−18^	6.64 × 10^−18^
10	1.90 × 10^−18^	3.31 × 10^−18^
20	1.18 × 10^−18^	2.30 × 10^−18^
50	1.94 × 10^−18^	1.64 × 10^−18^
100	6.31 × 10^−19^	8.56 × 10^−19^
1000	1.28 × 10^−19^	1.04 × 10^−19^

**Table 5:** Summary of chromium diffusion coefficients for conventional materials at a temperature of 1273 K.

	Material	D_Cr_ (m^2^·s^−1^)	Literature
T = 1273 K	Super304H steel	3.45 × 10^−18^	[31]
	TP304H steel	2.02 × 10^−18^	[31]
	Low carbon steel	2.62 × 10^−13^	[32]
	Low carbon steel	1.09 × 10^−15^	[32]
	Low carbon steel	1.16 × 10^−11^	[32]
	20Cr32Ni	9.14 × 10^−16^	[33]
	18Cr8Ni	1.83 × 10^−15^	[33]
	12CrMoV	4.57 × 10^−16^	[33]
	9CrMoV	8.06 × 10^−16^	[33]
	Ni-Fe	0.35 × 10^−16^	[34]
t = 250 h	Ni_3_Al	1.04 × 10^−16^	[35]

**Table 6:** Summary of titanium diffusion coefficients for conventional materials at a temperature of 1273 K.

	Material	D_Ti_ (m^2^·s^−1^)	Literature
1273 K	Ti/Nb	2.23 × 10^−14^	[36]
	Ti/Nb	3.28 × 10^−14^	[36]
	Ni3Al	3.43 × 10^−18^	[35]
	Ni3Al	8.00 × 10^−17^	[37]

## Data Availability

The data are available in a publicly accessible repository.
